# The Management of Desmoid Tumors: A Retrospective Study of 30 Cases

**DOI:** 10.1155/2020/9197216

**Published:** 2020-07-18

**Authors:** Yosr Zenzri, Yosra Yahyaoui, Lamia Charfi, Zahra Ghodhbani, Feryel Letaief, Mouna Ayadi, Amel Mezlini

**Affiliations:** ^1^Medical Oncology Department, Salah Azaiez Institute, Tunis, Tunisia; ^2^Pathology Department, Salah Azaiez Institute, Tunis, Tunisia

## Abstract

**Objectives:**

Desmoid tumor also called aggressive fibromatosis is a rare type of benign tumor. It is a mesenchymal malignancy without metastatic potential. The standard management is resection, but other options including observation may be discussed. Desmoid-type fibromatosis may occur throughout the body, but the abdominal wall is the most common site. The aim of our study was to assess the clinicoepidemiological profile, prognostic factors, and treatment outcome of desmoid tumors.

**Methods:**

A monocentric retrospective study was conducted over a period of 19 years between February 2000 and November 2019 at the oncology department of Salah Azaïz Institute. Our study concerns 30 patients with desmoid tumor. All data regarding patients were obtained from the medical record.

**Results:**

Thirty patients were included. The median age was 35 years with a female predominance (sex ratio = 0.07). A palpable mass was the most common complaint (*n* = 27). Median tumor size was 5 cm. The principal site of involvement was the abdominal wall (*n* = 14). Surgery was performed in 27 patients. The histopathology reports listed 14 (52%) cases with negative margins and 13 (48%) cases with positive margins. Radiation therapy was performed in 2 patients. One patient received tamoxifen. Local recurrence occurred in 11 patients. Two patients died of their desmoid tumor. Abdominal wall tumors have less risk of recurrence compared with other sites (*p*=0.047). Macroscopic margin involvement (R2) was the only prognostic factor influencing disease-free-survival (*p*=0.034).

**Conclusion:**

Desmoid tumors are aggressive tumors with a tendency for local recurrence. Abdominal wall tumors have less risk of recurrence. Macroscopic margin involvement was the only prognostic factor that affects disease-free-survival.

## 1. Introduction

Aggressive fibromatosis also called desmoid tumors (DTs) are soft tissue malignancies originating from fascial planes, connective tissues, and musculoaponeurotic structures of the muscles [1]. DTs are rare and constitute less than 3% of all soft tissue tumors and 0.03% of all neoplasms [2]. They are typically diagnosed in young adults with a female predominance [3].

DTs are a benign proliferation of myofibroblasts. This entity does not have the ability to metastasize. Although benign, these tumors can be locally invasive, causing pain or deformity that could make surgical removal more difficult.

The clinical behavior and natural history of aggressive fibromatosis remain unpredictable.

The symptoms depend on location and size of the tumor. The presentation can vary from asymptomatic to severe pain, swelling, deformity, and loss of function.

In fact, these tumors can be very aggressive with rapid growth and mass effect while others can be indolent and spontaneously regress [4].

The exact etiology of these tumors is unknown but trauma, hormonal, and genetic factors are considered to be correlated with the development of DTs [5].

Wide surgical excision remains the mainstay for resectable aggressive fibromatosis. But, alternative treatment modalities including radiation therapy, chemotherapy, tamoxifen, tyrosine kinase inhibitors, and nonsteroidal anti-inflammatory drugs may be used as a treatment for recurrent disease or as primary therapy to avoid mutilating surgical resection [6].

The aim of our study was to assess the clinicoepidemiological profile, prognostic factors and treatment outcome of DTs.

## 2. Materials and Methods

### 2.1. Study Design

A retrospective study including all consecutive patients with DTs was performed at a tertiary-level hospital (Salah Azaïz Institute) from February 2000 to November 2019. This study has been approved by the institution's Ethics Committee. Consent to participate was waivered do to the retrospective nature of the study. Data were collected and handled anonymously. The diagnosis of DTs was based on clinical, radiological, and histological criteria. Only patients with histologically confirmed aggressive fibromatosis who received their treatment at a study center were included. The histological diagnosis was made by the pathologists of the institute.

### 2.2. Data Collection

Data extracted as part of this retrospective analysis included age at diagnosis, gender, date of diagnosis, tumor size, primary site, surgical margins, recurrence rate, date of progression, time to progression, date of last follow-up, and survival status.

Lesions at the buttock, axilla, and groin were classified as extremity. Lesions at the neck, because of their small number in our study (*n* = 1), were considered with extremity lesions.

Margin was classified as gross positive (R2), microscopic positive (R1), or negative (R0).

Recurrence was defined as a lesion that histological examination has proven recurrent aggressive fibromatosis or a lesion that was deemed suspicious on imaging.

### 2.3. Statistical Analysis

The data were analyzed on the Statistical Package for the Social Sciences (SPSS) version 20 (IBM SPSS, Armonk, NY, USA). Results were expressed as mean ± standard deviation. Univariate comparisons between groups were performed using chi-squared tests for dichotomous variables or Fisher's test when appropriate. For continuous variables, independent samples *t* tests were used. *P* < 0.05 was considered to be significant.

Cumulative event rates were calculated using the method of Kaplan–Meier. Survival curves were compared using the log-rank test. Recurrence-free survival was determined as the time from diagnosis to either histology-proven or radiologic evidence of disease recurrence.

## 3. Results

The median age of the 30 patients at study inclusion was 35 years (range, 18–80 years). Patients with aggressive fibromatosis were predominantly female (*n* = 28; 93%). The average delay in consultation was 6 months. Three patients had history of a surgical procedure at the site of the tumor, while 2 patients recalled nonsurgical trauma at the site of tumor. None of the patients had history of familial adenomatous polyposis (FAP).

The diagnosis of aggressive fibromatosis was made during the first trimester of pregnancy in one patient. A palpable mass was the most common complaint (*n* = 27). Six patients were complaining of extreme pain. One patient present with a swelling and functional disability. DTs were located in the extremities (*n* = 9; 30%), abdominal cavity (*n* = 4; 13%), abdominal wall (*n* = 14; 47%), or thorax (*n* = 3; 10%). The median size of the tumor was 5 cm (range, 2.5–17.5 cm).

Eighteen patients (60%) received magnetic resonance imaging (MRI) examination, and nineteen (63%) had ultrasonography. The diagnosis was based on histological samples obtained by core-needle biopsy in 20 patients (67%).Characteristics of the 30 patients included in our study are detailed in https://www.ncbi.nlm.nih.gov/pmc/articles/PMC3568525/table/T1/Table 1.

**Figure 1:**
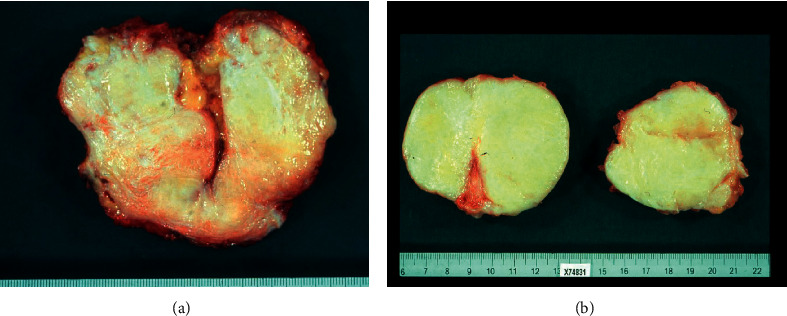
Macroscopically, the tumors were tenacious, yellowish white on the cut.

**Figure 2:**
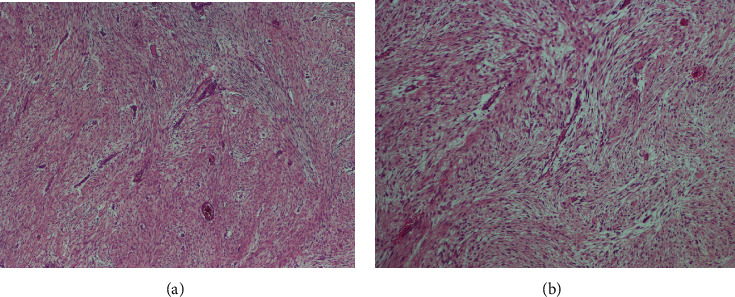
Interlacing bundles of fibroblasts separated by variable amounts of collagen in extra-abdominal fibromatosis. Regularly distributed blood vessels are conspicuous.

Wide surgical resection was performed in 27 patients (90%). In 14 cases, a wide (R0) resection, in 11, a marginal (R1) resection, and in 2 cases, an intralesional (R2) resection was achieved. Immunohistochemical analysis revealed that the tumor cells were strongly positive for beta-catenin in 10 patients (33%). Figures 1 and 2 illustrate the macroscopic and microscopic aspects of the desmoid tumors.

Two cases were managed by simple observation. Two patients with R1 resection underwent adjuvant radiation therapy. One patient with R2 resection received tamoxifen after surgery.

One patient developed an acute respiratory failure and died before starting any treatment.

Treatment characteristics are detailed in Table 2 (https://www.ncbi.nlm.nih.gov/pmc/articles/PMC3568525/table/T1/).

The median follow-up was 40 months. Local recurrence (LR) occurred in 11 (38%) patients within a median time interval of 21 months (range, 1–60 months). Of the 11 patients with LR, 6 had positive margins after initial surgery. Eight patients (73%) underwent repeat resection, 2 patients were lost to follow-up, and one patient developed bowel obstruction and died before resection. Two patients had adjuvant radiation therapy. Four of these 8 patients had a second relapse. An attempt at another resection was undertaken in three patients. One patient was lost to follow-up. One of these three patients developed a subsequent recurrence during follow-up.

**Figure 3:**
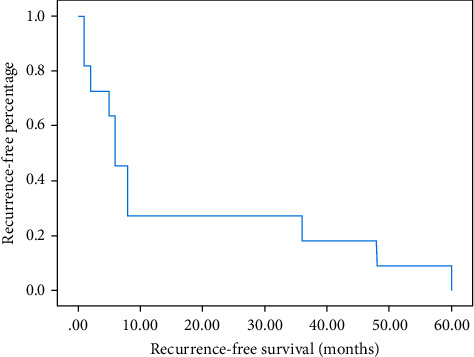
Recurrence-free survival analyzed by the Kaplan–Meier method.

The 1 and 3-year recurrence-free survival was 27 and 18%, respectively (Figure 3).

Abdominal wall tumors had less risk of recurrence compared with other sites (*p*=0.047).

**Figure 4:**
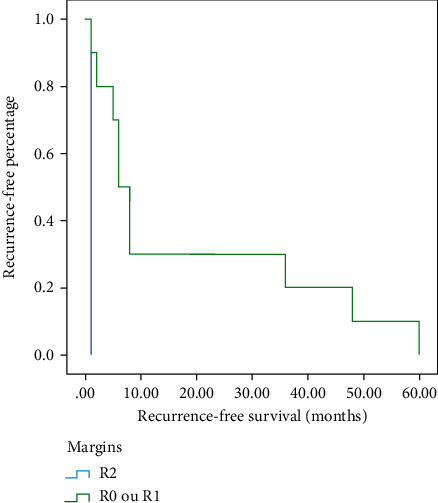
The Kaplan–Meier plot of local recurrence according to margin.

Macroscopic margin involvement (R2) was the only prognostic factor influencing disease-free-survival (*p*=0.034) (Figure 4).

In univariate analysis, age, history of trauma, and size of the tumor did not show a significant impact on local recurrence. The multivariate analysis could not be performed due to the limited number of patients.

## 4. Discussion

Aggressive fibromatosis is uncommon and accounts for only 0.03% of neoplasms and less than 3% of soft tissue tumors [2].They are described as a clonal fibroblastic proliferation in soft tissues with infiltrative growth [7]. They are locally aggressive tumors with a high rate of local recurrence after surgery but rarely metastasize. Indeed, they have unpredictable clinical course. DTs can appear in any anatomic location, frequently in the abdominal region: in our study, the tumor was located in the abdominal wall (47%), abdominal cavity (13%), extremities (30%), and thorax (10%). Other studies suggested a completely different distribution: abdominal wall (17.4%), abdominal cavity (10. 8%), and extra-abdominal (69.5%) [8]. The presentation can vary from asymptomatic to pain, deformity, swelling, or loss of function. The exact causes of DTs are largely unknown, but several factors may be involved. DTs usually occur in individuals between 15 and 60 years with a female preponderance [9]: 93% (*n* = 28) in our study and 67% in other series [10]. But, there is an equal incidence in males and females in DTs associated with FAP [11]. Pregnancy is associated with DTs due to high estrogenic levels [12]. DTs are related to trauma mainly surgical ones: 16% of our patients had a history of trauma. It seems to be one of the most important etiologic factors. Indeed, this can be explained by molecular connection between fibroproliferative disorders of mesenchymal tissue and wound healing processes [13].

DTs are mainly sporadic, but they can also be associated with familial infiltrative fibromatosis, hereditary desmoid disease, Gardner syndrome, and familial adenomatous polyposis .10 to 20% of DTs are associated with FAP while 10–30% of patients with FAP develop desmoid tumors [14, 15]. Colonoscopy should be done for patients with DTs especially if they are young or have intra-abdominal or abdominal wall tumor.

Histological examination of DTs revealed some characteristics: paucicellular proliferation of fibroblasts/myofibroblasts in a dense collagenous background, spindle cells with small and regular nuclei, pale eosinophilic cytoplasm, and acellular central area with increasing cellularity to the periphery [16]. The *β*-catenin positivity is caused by CTNNB1 mutation in chromosome 3p22. It is mainly found in sporadic DTs [17]. In contrast, DTs associated with FAP are caused by germline APC mutation in chromosome 5 (5q21e22) and then a somatic inactivation [18]. Nuclear positivity of *β*-catenin supports the diagnosis of DTs. Moreover, some studies suggested that a very strong *β*-catenin positivity is correlated with a high risk of recurrence [19]. But, nuclear *β*-catenin is not always specific, it can be positive in palmer/plantar fibromatosis, solitary fibrous tumor, Gardner fibroma, synovial sarcoma, and low-grade fibromyxoid sarcoma and not all DTs will stain for nuclear *β*-catenin: cytoplasmic staining is more sensitive but less specific [20].

MRI is the modality of choice for assessment of the nature and size of DTs. 60% of our patients has undergone this imaging. DTs show some features in MRI: they have stellar shape; they extend into the fascial planes and fat tissue in a sun-burst-like form. Besides, they are isointense on T1 and hyperintense on T2 [21].

In most series, local recurrence rates after surgical resection depend on margins. Indeed, these rates range from 80% to 10% when surgical margins are positive or negative, respectively [22]. In our study, 11 patients (38%) had local recurrence: 6 patients had PM and 5 patients had NM.

Macroscopic margin involvement (R2) was the only prognosis factor influencing the disease-free-survival (*p*=0.034).

Therefore, positive margins seem to be the most important indicator for an adjuvant treatment as radiation therapy.

Many studies showed the importance of radiotherapy. In a study published by Jelinek et al., adjuvant radiotherapy was the only significant prognostic factor for local control. The 5-year local control rate was 53% for patients who underwent surgery alone and 81% for patients who had surgery and radiation therapy (*p*=0.018) [23].

The effects of involved surgical margins after surgery remain unclear. PM were predictors for failure in some studies [24] but not in others [25].

In our study, abdomen wall tumors had less risk of recurrence compared with that in other sites (*p*=0.047). There was no recurrence in a series of 7 abdominal wall tumors published by Sutton and Thomas [26]. Shao et al. reported a recurrence rate of 5.5% in a large series of 42 abdominal wall tumors [27].

Other prognostic factors for recurrence have been identified, but they are still under investigation. In some studies, tumors larger than 8 cm were more likely to recur (*p*=0.021) [28]. Crago et al. discovered that tumors larger than 10 cm were more likely to relapse [10]. Age at diagnosis was a significant factor for recurrence in many studies. Crago et al. found more recurrence in patients younger than 26 years [10]. In our series, tumor size and age were not factors for local relapse.

There are multiple protocols to treat DTs: simple surveillance, surgery, chemotherapy radiation therapy, tyrosine kinase inhibitors, hormonal treatment, and nonsteroidal anti-inflammatory drugs.

Some series compared a simple surveillance protocol versus an active treatment and showed that the 5 years progression-free survival is almost the same (49.9% versus 58.6%) [29].

In other series, a spontaneous regression was observed in 20% of cases [30].

This result was in accordance with the wait-and-see method. However, when patients are symptomatic and experiencing rapid or life-threatening progression and the tumor is resectable with negative margins, surgery seems to be the best option [30]. But, mutilating surgical resection and function loss are the disadvantages of surgery.

Systemic treatment options for aggressive fibromatosis that are not amenable to surgery or radiation therapy comprise nonsteroidal anti-inflammatory drugs (NSAIDs), antihormonal therapies, tyrosine kinase inhibitors (TKIs), and chemotherapy.

Antihormonal therapies such as tamoxifen can be used alone or in association with NSAIDs as first-line treatment. There are some suggesting that higher doses (up to 120 mg/day) in combination with NSAIDs are more effective than tamoxifen alone [31].

COX-2 seems to play a role in the pathogenesis of DTs. NSAIDs has influence on the *β*-catenin pathway. NSAIDs that inhibit COX may be an alternative treatment. Indomethacin and sulindac were used in the treatment of DTs. As a result, partial and complete response was achieved in some nonrandomized retrospective studies [32]. But, the risk of cardiovascular events may be increased in patients receiving NSAIDs. This treatment should be avoided for some fragile patients.

The chemotherapeutic option can be the treatment of choice in cases of unresectable, aggressively growing and/or life-threatening tumors. Many chemotherapy protocols have been used. In the pediatric population, weekly administration of methotrexate and vinblastine has been evaluated.

Skapek et al. found that this regimen was well tolerated, and the response rate was 19% in a cohort of 28 patients [22]. But, there is no prospective data for this combination in adults. Antracyclines appeared to have a higher response rate. This regimen is administrated for 6 to 8 cycles [33]. Pegylated liposomal doxorubicin at a dose of 50 mg/m^2^ every 4 weeks showed acceptable toxicity and a significant activity [34]. Tyrosine kinase inhibitors (imatinib and sorafenib) have demonstrated activity in the treatment of DTs because of the PDGFRs expressed in tumor stroma.

In a series published by Gounder et al., Sorafenib was administered at 400 mg oral daily. There was a 30% reduction of the tumor size in 92% of the patients [35]. Penel et al. evaluated the efficacy of Imatinib in patients with unresectable and progressive symptomatic DTs. This treatment showed that 83% of patients had stable disease; 3% had complete response rate and 9% had partial response rate [36].

Due to the unpredictable behavior of DTs, such as long extended periods of stable disease and even rapid progression, treatment must be individualized to improve the efficiency of local tumor control and protect the quality of life. Consequently, the application of a multidisciplinary assessment along with multimodality treatment shapes the foundation of care for the related patients.

## 5. Conclusions

DTs are aggressive mesenchymal tumors with a tendency for local recurrence. Abdominal wall tumors have less risk of recurrence. Macroscopic margin involvement was the only prognostic factor that affects disease-free-survival. A multidisciplinary approach is mandatory. Treatment recommendations including surgery, radiation therapy, and systemic therapy are all evolving.

## Figures and Tables

**Table 1:** Characteristics of patients.

Treatment	Frequency	%
*Sex*		
Male	2	7
Female	28	93

*Median Age* = *35 years*		
History of a surgical procedure at the site of the tumor	3	10
History of nonsurgical trauma at the site of tumor	2	7
History of pregnancy in females with tumor	1	3

*Complaints*		
Palpable mass	27	90
Pain	6	20
Swelling and functional disability	1	3

*Location of primary tumor*		
Abdominal wall	14	47
Extremities	9	30
Abdominal cavity	4	13
Thorax	3	10

*Median size of the tumor* = 5 cm		
Imaging ultrasonography	19	63
MRI	18	60
CT scan	12	40
Biopsy	20	67

**Table 2:** Treatment characteristics.

Treatment	Frequency	%
Wide surgical resection	27	90
*Surgical margins*		
R0	14	52
R1	11	41
R2	2	7
Beta-catenin	10	33
Observation	2	7
Adjuvant radiation therapy	2	7
Tamoxifen	1	3

## Data Availability

The data that support the findings of this study are available from the corresponding author upon reasonable request.
